# The causal relationship between gut microbiota and lymphoma: a two-sample Mendelian randomization study

**DOI:** 10.3389/fimmu.2024.1397485

**Published:** 2024-05-07

**Authors:** Biyun Li, Yahui Han, Zhiyu Fu, Yujie Chai, Xifeng Guo, Shurui Du, Chi Li, Dao Wang

**Affiliations:** ^1^ Department of Pediatric Hematology Oncology, The First Affiliated Hospital of Zhengzhou University, Zhengzhou, Henan, China; ^2^ Department of Pediatric Surgery, The First Affiliated Hospital of Zhengzhou University, Zhengzhou, Henan, China

**Keywords:** gut microbiota, Mendelian randomization, Hodgkin lymphoma, non-Hodgkin lymphoma, causal effect

## Abstract

**Background:**

Previous studies have indicated a potential link between the gut microbiota and lymphoma. However, the exact causal interplay between the two remains an area of ambiguity.

**Methods:**

We performed a two-sample Mendelian randomization (MR) analysis to elucidate the causal relationship between gut microbiota and five types of lymphoma. The research drew upon microbiome data from a research project of 14,306 participants and lymphoma data encompassing 324,650 cases. Single-nucleotide polymorphisms were meticulously chosen as instrumental variables according to multiple stringent criteria. Five MR methodologies, including the inverse variance weighted approach, were utilized to assess the direct causal impact between the microbial exposures and lymphoma outcomes. Moreover, sensitivity analyses were carried out to robustly scrutinize and validate the potential presence of heterogeneity and pleiotropy, thereby ensuring the reliability and accuracy.

**Results:**

We discerned 38 potential causal associations linking genetic predispositions within the gut microbiome to the development of lymphoma. A few of the more significant results are as follows: Genus *Coprobacter* (OR = 0.619, 95% CI 0.438–0.873, *P* = 0.006) demonstrated a potentially protective effect against Hodgkin’s lymphoma (HL). Genus *Alistipes* (OR = 0.473, 95% CI 0.278–0.807, *P *= 0.006) was a protective factor for diffuse large B-cell lymphoma. Genus *Ruminococcaceae* (OR = 0.541, 95% CI 0.341–0.857, *P *= 0.009) exhibited suggestive protective effects against follicular lymphoma. Genus *LachnospiraceaeUCG001* (OR = 0.354, 95% CI 0.198–0.631, *P *= 0.0004) showed protective properties against T/NK cell lymphoma. The *Q* test indicated an absence of heterogeneity, and the MR-Egger test did not show significant horizontal polytropy. Furthermore, the leave-one-out analysis failed to identify any SNP that exerted a substantial influence on the overall results.

**Conclusion:**

Our study elucidates a definitive causal link between gut microbiota and lymphoma development, pinpointing specific microbial taxa with potential causative roles in lymphomagenesis, as well as identifying probiotic candidates that may impact disease progression, which provide new ideas for possible therapeutic approaches to lymphoma and clues to the pathogenesis of lymphoma.

## 1 Introduction

Lymphoma constitutes a category of neoplastic disorders originating from the lymphohematopoietic system. It can be classified into two categories—Hodgkin’s lymphoma (HL) and non-Hodgkin’s lymphoma (NHL)—based on the morphological characteristics of the tumor cells (1). In Western countries, lymphomas account for approximately 4% of newly diagnosed malignancies, ranking them as the fifth most prevalent type of cancer (2, 3). As per international cancer data trends, the incidence of lymphoma has been continuously rising (4, 5). The cause of lymphoma is not entirely clear, although previous studies have suggested a possible link to factors such as smoking (6), alcohol consumption (7), obesity (8), viral infections (9, 10), ionizing radiation exposure (11), chemical exposure (12), autoimmune diseases, or immune dysfunction (13). Moreover, there is mounting evidence that the gut microbiota significantly affect lymphoma pathogenesis, treatment response, and prognosis (14).

The various microorganisms and their ecology in the human gastrointestinal tract constitute the complex gut microbiota (15). Previous research have shown that the gut microbiota appears to influence human pathophysiological phenomena, encompassing immunity modulation, metabolic regulation, and inflammatory response mechanisms (16, 17). The perturbation of gut microbiota, commonly referred to dysbiosis, is increasingly considered as a potential precursor, facilitator, or even an instigating factor for a multitude of malignancies (18). Gut microbiota dysbiosis has been consistently noted across numerous lymphoma investigations, thereby giving rise to the conceptual framework known as the “microbiota–gut–lymphoma axis” (19)—for instance, gastric mucosa-associated lymphoid tissue (MALT) lymphoma in the stomach is strongly associated with infection with *Helicobacter pylori* (20). SE Yoon and colleagues found that patients diagnosed with diffuse large B-cell lymphoma (DLBCL) exhibit significantly reduced α-diversity compared to healthy subjects, coupled with a marked elevation in the abundance of *Enterobacteriaceae* family bacteria relative to those seen in healthy controls (21). The gut microbiome has also emerged as a promising diagnostic biomarker. A study conducted by Z Shi and colleagues identified the role of gut microbiota that was illuminated in terms of its utility for diagnosing natural killer/T-cell (NK/T cell) lymphoma (22). In addition, the gut microbiome might play a regulatory role in modulating the effectiveness of immunotherapy for lymphoma. A research found that administering broad-spectrum antibiotics prior to CD19-targeted chimeric antigen receptor T-cell (CAR-T) treatment resulted in unfavorable outcomes (23). In the context of hematopoietic stem cell transplantation (HSCT) among lymphoma patients, research had revealed a correlation between the proliferation of *Lactobacillus* species with the exacerbation or worsening of graft-*versus*-host disease (GVHD) (24). Previous studies have indicated that the gut microbiota plays a regulatory role in the effectiveness of cancer immunotherapies, and there is an opportunity for targeted microbiota to enhance anti-cancer efficacy while reducing toxicity in microbial therapies, which is crucial for developing personalized cancer treatment strategies (25).

The majority of the aforementioned studies have adopted a case–control design, which inherently carries limitations in establishing a definitive causal relationship between the exposure and the outcome under investigation. Furthermore, compared with traditional observational studies, the link is less susceptible to potential confounding factors, including environmental factors, dietary habits, and lifestyle. Nevertheless, establishing causal connections would improve our understanding of the gut microbiota’s role in lymphoma pathogenesis and have the potential to guide tailored microbiota-based interventions against various forms of lymphoma in clinical settings.

Mendelian randomization (MR) is an increasingly adopted natural randomization method that utilizes genetic variation as instrumental variables (IVs) (26). It follows Mendelian randomization second law, which identifies genetic variations at conception and follows a pattern akin to random allocation, and is widely used in studies of causality in disease etiology (27). In addition, compared with traditional observational studies, MR effectively reduces biases arising from confounding factors or reverse causality, ensuring greater reliability and validity of experimental findings (28). Recently, MR analyses have been implemented to explore causal links between the gut microbiota and various types of cancers (29). This study employs MR to analyze the potential causal effects of gut microbiota composition on lymphoma given the uncertainty of the causal relationship between the two. The aim is to establish a robust theoretical framework that can facilitate further investigation to the development of lymphoma.

## 2 Materials and methods

### 2.1 Data sources

Gut microbe-related genome-wide association studies (GWAS) data were meticulously retrieved from the esteemed MiBioGen Global Consortium, an expansive multi-ethnic study that orchestrates a grand-scale GWAS. The data was derived from an aggregate of 18,340 participant, including cohorts from Germany, Canada, Denmark, Israel, and The Netherlands among others (30). We eliminated 15 bacterial taxa lacking clear taxonomic identification along with one duplicative bacterial taxon entry (31). A set of 195 bacterial taxa stood out as the core elements underpinning the exposure variables in our subsequent MR analyses.

The malignant lymphoma GWAS databases were conveniently accessible *via* the FinnGen project’s online portal. Among these, the GWAS dataset pertaining to HL comprised 846 cases juxtaposed against an impressive backdrop of 324,650 controls. Similarly, the GWAS data linked with NHL featured an extensive array of subtypes: follicular lymphoma (FL) incorporating 1,181 cases, DLBCL with 1,050 cases, mature T/NK-cell lymphomas documented with 363 cases, and a collective category for other and unspecified NHL types accounting for 1,171 cases.

All the aforementioned GWAS datasets hold the virtue of being publicly accessible and can be effortlessly downloaded from the OPEN GWAS web platform. For clarity and reference, the specific dataset details employed in our Mendelian randomization (MR) analysis have been systematically presented in **Table 1**.

**Table 1:** Data information.

Datasets	Ancestry	Sample size	NSNP	Consortium	Web source
Gut microbiota	European	18,340	122,110	MiBioGen	https://mibiogen.gcc.rug.nl/
HL	European	846/324,650	21,304,278	FinnGen	https://storage.googleapis.com/finngen-public-data-r10/summary_stats/finngen_R10_CD2_HODGKIN_LYMPHOMA_EXALLC.gz
DLBCL	European	1,050/314,193	21,303,852	FinnGen	https://storage.googleapis.com/finngen-public-data-r10/summary_stats/finngen_R10_C3_DLBCL_EXALLC.gz
FL	European	1,181/324,650	21,304,293	FinnGen	https://storage.googleapis.com/finngen-public-data-r10/summary_stats/finngen_R10_CD2_FOLLICULAR_LYMPHOMA_EXALLC.gz
T/NK cell lymphoma	European	363/324,650	21,304,264	FinnGen	https://storage.googleapis.com/finngen-public-data-r10/summary_stats/finngen_R10_CD2_TNK_LYMPHOMA_EXALLC.gz
Other and unspecified types of NHL	European	1,171/324,650	21,304,287	FinnGen	https://storage.googleapis.com/finngen-public-data-r10/summary_stats/finngen_R10_CD2_NONHODGKIN_NAS_EXALLC.gz

HL, Hodgkin’s lymphoma; NHL, Non-Hodgkin’s Lymphoma; DLBCL, diffuse large B-cell lymphoma; FL, follicular lymphoma.

### 2.2 Selection of instrumental variables

This study capitalizes on single-nucleotide polymorphisms (SNPs) as IVs in its analytical framework. For these IVs to be rigorously employed within MR analyses, they must satisfy three basic conditions as described below:

(1) Relevance: Acknowledging that the limitation in the number of SNPs within the gut microbiome reached the genome-wide threshold of statistical significance (*P* < 5 × 10^-8^), we adopted a *p*-value <1 × 10^-5^ in selecting SNPs associated with risk factors to obtain comprehensive and reliable results (32). To ensure the independence of IVs, we employed a linkage disequilibrium (LD) threshold where *r*² was set to be less than 0.001, coupled with clumping distances exceeding 10,000 kb. This strategy is designed to filter out genetic variants with a weaker capacity to elucidate exposure, which might potentially influence the results. Utilizing established methods, we used the equation *R* ^2^ = 2 × eaf × (1 − eaf) × beta^2^ to calculate the proportion of exposed variation attributable to each SNP. In addition, the *F*-statistic between each SNP and gut microbiota was calculated using the equation *F* = *R* ^2^ × (N − 2)/(1 − *R* ^2^) (33, 34). In doing so, SNPs with *F <*10 were discarded, considering them as weak instruments. This multi-level filtering process serves to ensure a strong and meaningful association between the retained SNPs and the gut microbiota under investigation.
(2) Independence: To investigate the potential associations between each SNP and confounders, we used the online Phenoscanner platform (available at http://www.phenoscanner.medschl.cam.ac.uk). We excluded SNPs with confounding factors associated with lymphoma (e.g., smoking, alcohol, body mass index, virus infections, immune abnormalities, chemical exposure, and ionizing radiation exposure). The Mendelian Randomization Pleiotropy Residual Sum and Outlier (MR-PRESSO) analysis serves as a powerful tool adept at detecting and differentiating outliers and SNPs exhibiting pleiotropic effects. In this analytical pipeline, the MR-PRESSO test calculates individual SNP and global test, respectively. The *p*-values for individual SNPs are sorted in ascending order and eliminated one by one. Afterward, a new MR-PRESSO global test is performed on the residual SNPs to reassess the overall horizontal multidirectionality. This recursive process continued until the global test returned a non-significant *p*-value (*p* > 0.05) (35).
(3) Exclusivity: To validate the unidirectional nature of the causal pathway, the MR-Steiger method was used to rigorously measure the directional estimates of causality (36). SNPs with incorrect direction were excluded.

### 2.3 MR analyses

We conducted MR analyses between gut microbiota and lymphoma. If a single independent variable (IV) representing a specific gut microbiological profile was associated with a lymphoma subtype, we used the Wald’s ratio test (37). When dealing with features characterized by multiple IVs, we applied a suite of five widely recognized MR methodologies: the inverse variance weighted (IVW) test (38), the MR-Egger regression (39), the weighted median estimator (WME) (40), simple mode, and weighted mode (41).

The IVW approach is a meta-analytic tool that integrates the Wald ratio estimates derived from each SNP analysis by summing their estimates with inverse variance weighting (42). The MR-Egger regression was grounded on the no-error-of-measurement (NOME) assumption, using an intercept term to examine the presence of potential pleiotropic effects (39). Moreover, the WME and weighted mode approaches allow for the flexible estimation of causality, even though half of the IVs may be void. Relative to the MR-Egger approach, these two methods help to improve the precision of the study results (43). Simultaneously, we conducted supplementary analyses using both weighted mode and simple mode techniques to bolster the reliability of the primary IVW method results (44).

In summary, the IVW model assumes the central role as the principal analytical strategy when there is no heterogeneity and horizontal pleiotropy. However, when heterogeneity becomes evident, findings derived from the WME approach are deliberated upon. Should there be indications of horizontal pleiotropy, the MR-Egger regression supersedes as the main method of analysis. The association between gut microbial composition and lymphoma risk was quantified using odds ratios (OR) and their corresponding 95% confidence intervals (CI), where *p <*0.05 was considered statistically significant. Moreover, in ensuring robustness against false positives due to multiple testing, we stringently applied the Bonferroni correction to establish statistically adjusted significance thresholds at each taxonomic stratum. These levels were respectively set at phylum level (*α* = 0.05/9), class level (*α* = 0.05/15), order level (*α* = 0.05/20), family level (*α* = 0.05/32), and genus level (*α* = 0.05/119). Statistically significant *P*-values from MR analyses were interpreted as *prima facie* or suggestive evidence of a causal association if they were above the adjusted threshold.

### 2.4 Sensitivity analysis

We conducted pleiotropy analyses, leave-one-out (LOO) analysis, and heterogeneity tests to evaluate and mitigate the effects of uncertainty in the models. Horizontal pleiotropy—a phenomenon where a genetic variant influences multiple traits beyond the primary outcome—was rigorously measured using the MR-Egger approach. Notably, the presence of horizontal pleiotropy was suggested if MR-Egger analysis revealed a statistically significant intercept. Importantly, MR-Egger methodology permits the accommodation of pleiotropic genetic variants while still enabling the estimation of unbiased causal effects even when directional pleiotropy or substantial heterogeneity exists (45). Cochran’s *Q* test quantifies the degree of inconsistency between the chosen SNPs and, if the result is statistically significant, indicates model heterogeneity (46). Based on this finding, we tend to use random effects IVW for analyses where there is significant heterogeneity. A fixed-effects model was adopted instead (47). Additionally, we performed a LOO analysis, which is used to check whether individual SNPs disproportionately affect the overall estimate. This is done by systematically removing each SNP in succession and then reapplying the MR method to the residual data to examine the robustness of the estimated causal effects and ensure the stability of our findings (48).

### 2.5 Data visualization and statistical software

We plotted forest plots of the overall causal estimates based on the results of the IVW method as well as scatter plots and LOO forest plots for each causal relationship to illustrate the collective contribution of these SNPs. All statistical analyses were executed using the R software packages “TwoSampleMR” (49) and “MR-PRESSO” (35), which ensured a robust and comprehensive assessment of the data.

## 3 Results

### 3.1 Selection of IVs

**Figure 1:**
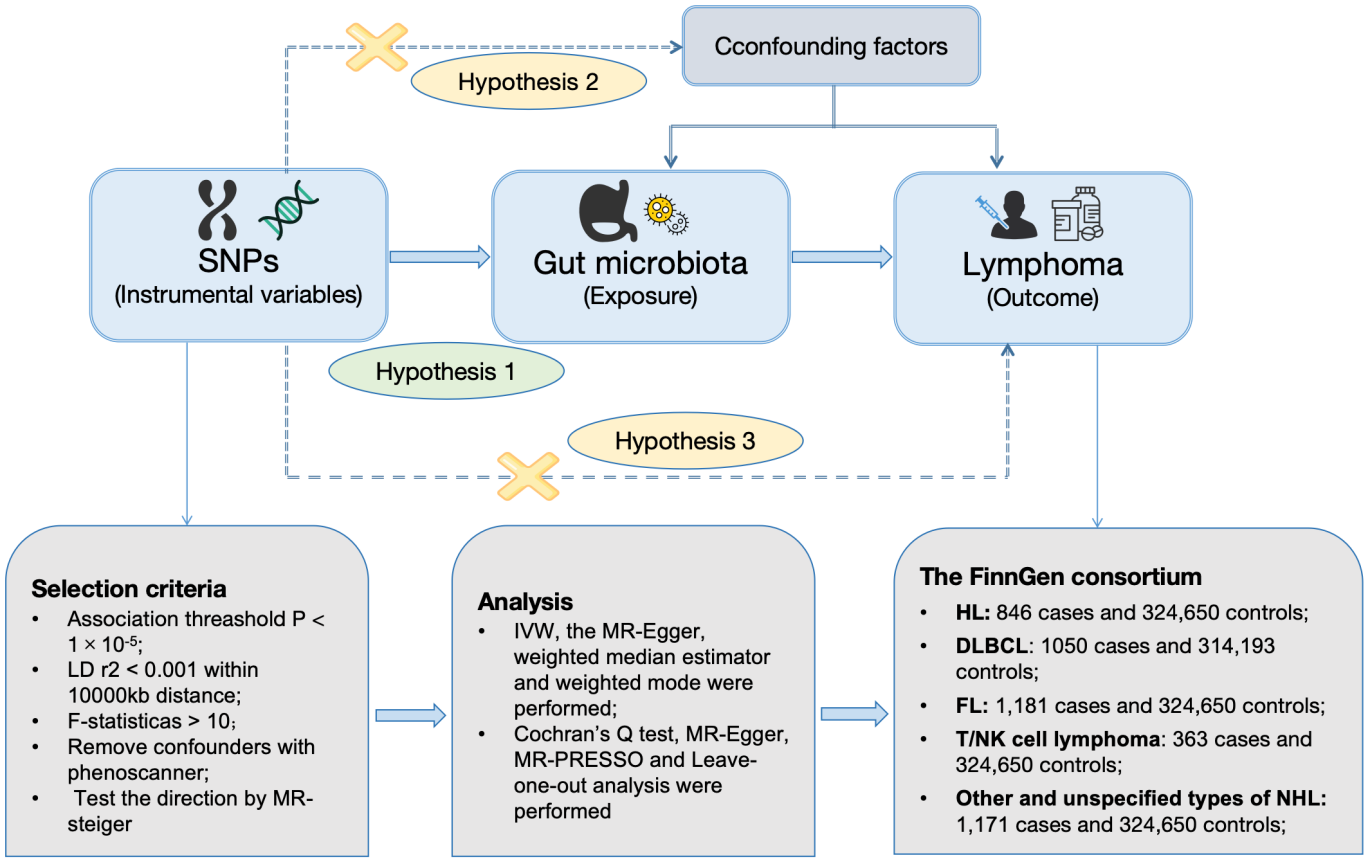
Workflow of MR design. HL, Hodgkin’s lymphoma; NHL, non-Hodgkin’s lymphoma; DLBCL, diffuse large B-cell lymphoma; FL, follicular lymphoma; MR, mendelian randomization; SNPs, single-nucleotide polymorphisms; IVW, inverse-variance weighted; WME, weighted median estimator; MR-PRESSO, Mendelian randomization pleiotropy residual sum and outlier; LD, linkage disequilibrium.

**Figure 1** was constructed to visually reflect the relationship between SNPs (IVs), risk factor (gut microbiota), and outcome (lymphoma). First, SNPs significantly associated with the gut microbiota were selected. We conducted a thorough screening using the PhenoScanner to exclude SNPs that may be associated with lymphoma risk-related confounders (**Supplementary Table S1**). Subsequently, we ascertained the calculated *F*-statistics >10 for all IVs (**Supplementary Table S2**), and the results of MR-PRESSO suggested that there was no significant pleiotropy in this study (**Table 2**). Additionally, through the application of MR-Steiger analysis, we confirmed that none of the SNPs exhibited reversed causality (**Supplementary Table S3**). After a rigorous screening of IVs, the remaining 2,548 eligible SNPs were included in the subsequent analyses. The IVs after harmonization are listed in **Supplementary Table S4**.

**Table 2:** Sensitivity analysis of the causal association between gut microbiota and lymphoma.

Bacterial taxa (exposure)	Lymphoma (outcome)	No. of SNPs	Heterogeneity		Pleiotropy		MR-PRESSO			MR Steiger
			Cochran *Q* test		MR-Egger					
			*Q*-value	*P*	Intercept	*P*	MR analysis causal estimate	SD	*P*	
Genus *Coprobacter*	HL	12	10.607	0.477	0.006	0.941	-0.480	0.172	0.490	True
Genus *Oscillospira*	HL	7	3.582	0.733	-0.134	0.291	0.567	0.211	0.737	True
Genus *Gordonibacter*	HL	11	7.709	0.657	0.026	0.766	0.268	0.119	0.674	True
Class Mollicutes	HL	11	11.203	0.342	0.045	0.558	-0.539	0.247	0.362	True
Phylum Tenericutes	HL	11	11.203	0.342	0.045	0.558	-0.539	0.247	0.353	True
Genus *CandidatusSoleaferrea*	HL	9	3.492	0.900	-0.020	0.915	-0.487	0.127	0.912	True
Genus *Eggerthella*	HL	10	9.789	0.368	-0.111	0.247	-0.430	0.181	0.386	True
Family *Bifidobacteriaceae*	HL	10	9.075	0.430	0.055	0.083	0.641	0.285	0.497	True
Order Bifidobacteriales	HL	10	9.075	0.430	0.055	0.083	0.641	0.285	0.453	True
Genus *Intestinimonas*	HL	16	10.262	0.803	-0.055	0.258	-0.464	0.162	0.816	True
Genus *Coprobacter*	DLBCL	12	6.380	0.701	-0.021	0.765	0.312	0.127	0.792	True
Genus *Alistipes*	DLBCL	12	4.490	0.953	-0.063	0.443	-0.748	0.174	0.950	True
Genus *Bilophila*	DLBCL	13	17.802	0.122	-0.045	0.659	0.575	0.267	0.126	True
Genus *RuminococcaceaeUCG011*	DLBCL	7	0.864	0.990	0.065	0.507	-0.333	0.055	0.992	True
Family *Desulfovibrionaceae*	DLBCL	10	6.380	0.701	0.051	0.271	0.457	0.195	0.745	True
Class Actinobacteria	FL	15	12.838	0.539	0.067	0.141	0.419	0.194	0.551	True
Family *Peptostreptococcaceae*	FL	13	9.429	0.666	0.037	0.298	-0.402	0.166	0.67	True
Genus *Alistipes*	FL	12	13.998	0.233	0.094	0.277	-0.571	0.289	0.268	True
Family *Rhodospirillaceae*	FL	14	9.556	0.730	0.138	0.050	-0.322	0.128	0.738	True
Genus *RuminococcaceaeNK4A214group*	FL	13	15.261	0.227	-0.028	0.639	-0.615	0.235	0.26	True
Genus *Haemophilus*	FL	9	5.444	0.709	-0.003	0.954	-0.353	0.142	0.718	True
Genus *Slackia*	FL	6	10.098	0.073	-0.398	0.042	-0.636	0.297	0.135	True
Order Pasteurellales	FL	14	9.156	0.761	0.029	0.438	-0.292	0.12	0.8	True
Family *Pasteurellaceae*	FL	14	9.15	0.761	0.029	0.438	-0.292	0.12	0.787	True
Genus *Catenibacterium*	FL	4	2.213	0.529	0.429	0.283	0.37	0.158	0.56	True
Genus *LachnospiraceaeUCG001*	T/NK cell lymphoma	13	11.158	0.515	0.006	0.957	-1.04	0.285	0.597	True
Order Methanobacteriales	T/NK cell lymphoma	10	2.623	0.977	-0.048	0.752	-0.555	0.120	0.977	True
Family *Lactobacillaceae*	T/NK cell lymphoma	9	1.956	0.982	0.020	0.832	-0.649	0.14	0.981	True
Genus *ChristensenellaceaeR.7group*	T/NK cell lymphoma	9	3.457	0.902	0.060	0.626	-1.03	0.33	0.927	True
Genus *Ruminococcus1*	T/NK cell lymphoma	10	9.033	0.434	-0.010	0.918	-0.815	0.412	0.458	True
Genus *RuminococcaceaeUCG014*	T/NK cell lymphoma	11	7.739	0.654	0.044	0.562	-0.886	0.314	0.707	True
Class Methanobacteria	T/NK cell lymphoma	10	2.623	0.977	-0.048	0.752	-0.555	0.120	0.985	True
Genus *Lactobacillus*	T/NK cell lymphoma	8	1.097	0.993	0.017	0.853	-0.69	0.114	0.992	True
Order Bacillales	Other and unspecified types of NHL	8	6.120	0.526	0.077	0.969	-0.281	0.117	0.538	True
Genus *Sutterella*	Other and unspecified types of NHL	12	14.559	0.204	-0.010	0.888	0.467	0.238	0.221	True
Genus *Slackia*	Other and unspecified types of NHL	6	5.112	0.402	-0.117	0.454	-0.442	0.212	0.518	True
Family *Defluviitaleaceae*	Other and unspecified types of NHL	11	4.650	0.875	0.024	0.718	0.424	0.122	0.923	True
Order Clostridiales	Other and unspecified types of NHL	14	14.297	0.353	0.025	0.720	0.509	0.236	0.419	True

HL, Hodgkin’s lymphoma; NHL, non-Hodgkin’s lymphoma; FL, follicular lymphoma; DLBCL, diffuse large B-cell lymphoma; MR, mendelian randomization; SNPs, single-nucleotide polymorphisms; MR-PRESSO, Mendelian Randomization Pleiotropy Residual Sum and Outlier; SD, standard deviation.

### 3.2 MR analysis results

**Figure 2:**
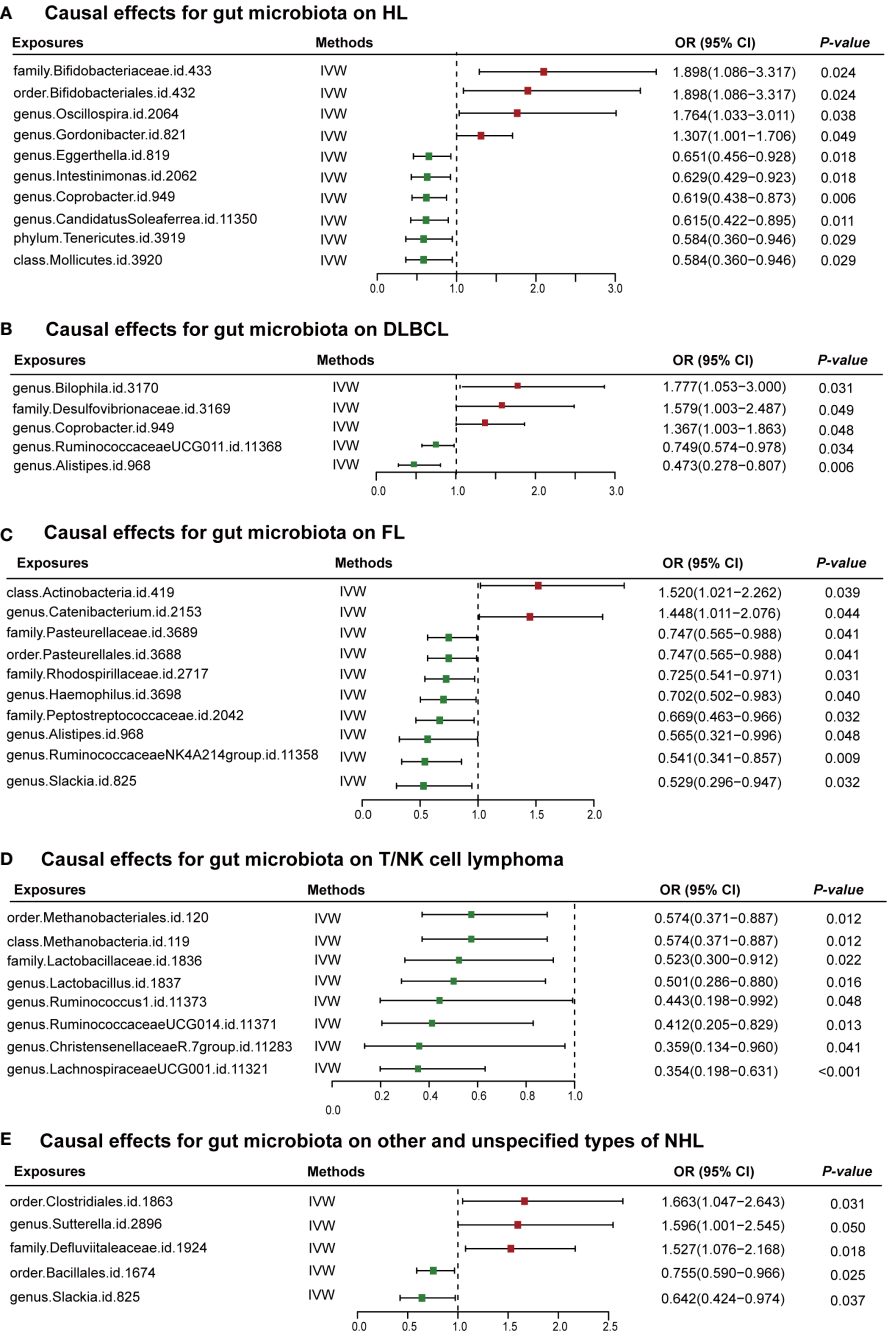
MR results and its forest plot. **(A)** Causal effects for gut microbiota on HL; **(B)** Causal effects for gut microbiota on DLBLC; **(C)** Causal effects for gut microbiota on FL; **(D)** Causal effects for gut microbiota on T/NK cell lymphoma; **(E)** Causal effects for gut microbiota on other and unspecified types of NH. HL, Hodgkin’s lymphoma; NHL, non-Hodgkin’s lymphoma; DLBCL, diffuse large B-cell lymphoma; FL, follicular lymphoma; OR, odds radio; 95% CI, 95% confidence interval; IVW, inverse-variance weighted.

The MR analysis using IVW identified 35 gut microbiota genera linked to lymphoma risk (**Figure 2**; **Supplementary Table S5**). Scatter plots corresponding to each causal relationship are provided in **Supplementary Figure S1** for further illustration.

In this study, 10 genetically inferred gut microbiota taxa were found to exhibit significant associations with HL. Notably, family *Bifidobacteriaceae* (OR = 1.898, 95% CI 1.086–3.317, *P* = 0.024), order Bifidobacteriaceae (OR = 1.898, 95% CI 1.086–3.317, *P* = 0.024), genus *Oscillospira* (OR = 1.764, 95% CI 1.033–3.011, *P* = 0.038), and genus *Gordonibacter* (OR = 1.307, 95% CI 1.001–1.706, *P* = 0.049) showed suggestive evidence of increasing the risk of HL development. On the contrary, phylum Tenericutes (OR = 0.584, 95% CI 0.360–0.946, *P* = 0.029), class Mollicutes (OR = 0.584, 95% CI 0.360–0.946, *P* = 0.029), genus *CandidatusSoleaferrea* (OR = 0.615, 95% CI 0.422–0.895, *P* = 0.011), genus *Coprobacter* (OR = 0.619, 95% CI 0.438–0.873, *P* = 0.006), genus *Intestinimonas* (OR = 0.629, 95% CI 0.429–0.923, *P* = 0.018), and genus *Eggerthella* (OR = 0.651, 95% CI 0.456–0.928, *P* = 0.0176) demonstrated a potentially protective role against HL incidence.

For DLBCL, MR analyses using the IVW method showed causal relationships with five bacterial taxa. Genus *Bilophila* (OR = 1.777, 95% CI 1.053–3.000, *P* = 0.031), family *Desulfovibrionaceae* (OR = 1.579, 95% CI 1.003–2.487, *P* = 0.049), and genus *Coprobacter* (OR = 1.367, 95% CI 1.003–1.863, *P* = 0.048) displayed a statistically suggestive association with elevated risks of DLBCL. On the contrary, genus *RuminococcaceaeUCG011* (OR = 0.749, 95% CI 0.574–0.978, *P* = 0.034) and genus *Alistipes* (OR = 0.473, 95% CI 0.278–0.807, *P* = 0.006) showed a negatively correlated relationship with the risk of developing DLBCL.

For FL, MR analyses using the IVW method showed causal relationships with 10 bacterial taxa. Two of those were positively correlated with an elevated risk of FL, class Actinobacteria (OR = 1.520, 95% CI 1.021–2.262, *P* = 0.039) and genus *Catenibacterium* (OR = 1.448, 95% CI 1.011–2.076, *P* = 0.044). On the contrary, family *Pasteurellaceae* (OR = 0.747, 95% CI 0.565–0.988, *P* = 0.041), order Pasteurellales (OR = 0.747, 95% CI 0.565–0.988, *P* = 0.041), genus *Alistipes* (OR = 0.565, 95% CI 0.321–0.996, *P* = 0.0484), genus *Haemophilus* (OR = 0.703, 95% CI 0.502–0.983, *P* = 0.040), genus *Slackia* (OR = 0.529, 95% CI 0.296–0.947, *P* = 0.032), family *Peptostreptococcaceae* (OR = 0.669, 95% CI 0.463–0.966, *P* = 0.032), family *Rhodospirillaceae* (OR = 0.725, 95% CI 0.541–0.970, *P* = 0.031), and genus *Ruminococcaceae* (OR = 0.541, 95% CI 0.341–0.857, *P* = 0.009) showed suggestive protective effects against FL.

Utilizing the IVW approach, a collective of eight bacterial taxa within the gut microbiome were identified to exhibit a statistically significant negative correlation with the onset and progression of T/NK cell lymphoma. They were genus *Ruminococcus1* (OR = 0.443, 95% CI 0.198–0.992, *P* = 0.048), genus *ChristensenellaceaeR.7group* (OR = 0.359, 95% CI 0.134–0.960, *P* = 0.041), family *Lactobacillaceae* (OR = 0.523, 95% CI 0.300–0.917, *P* = 0.022), genus *Lactobacillus* (OR = 0.501, 95% CI 0.286–0.880, *P* = 0.016), genus *RuminococcaceaeUCG014* (OR = 0.412, 95% CI 0.205–0.829, *P* = 0.013), order Methanobacteriales (OR = 0.574, 95% CI 0.371–0.887, *P* = 0.012), class Methanobacteria (OR = 0.574, 95% CI 0.371–0.887, *P* = 0.012), and genus *LachnospiraceaeUCG001* (OR = 0.354, 95% CI 0.198–0.631, *P* = 0.0004).

Regarding other and unspecified types of NHL, the application of the IVW method in MR analyses revealed significant causal linkages with a select group of five bacterial taxa. Genus *Sutterella* (OR = 1.600, 95% CI 1.001–2.545, *P* = 0.0497), order Clostridiales (OR = 1.663, 95% CI 1.047–2.643, *P* = 0.031), and family *Defluviitaleaceae* (OR = 1.527, 95% CI 1.076–2.168, *P* = 0.018) revealed a trend towards heightened risk. On the contrary, order Bacillales (OR = 0.755, 95% CI 0.590–0.966, *P* = 0.025) and genus *Slackia* (OR = 0.642, 95% CI 0.424–0.974, *P* = 0.0373) displayed a protective effect.

However, despite the statistical significance described above, these observed associations did not meet the strict thresholds imposed by the Bonferroni correction and therefore lost statistical significance after adjustment.

### 3.3 Sensitivity analysis results

We conducted several rigorous sensitivity analyses and found no factors that significantly affected the robustness of the findings, with the detailed outcomes summarized in **Table 2**. The LOO test found no outliers, suggesting stable results (**Supplementary Figure S2**). As mentioned above, the results of MR-Egger suggested that no significant horizontal pleiotropy was found in this study (**Supplementary Table S6**). Sagittarius conclusions were obtained in the MR-PRESSO test (**Supplementary Table S7**). Furthermore, no significant heterogeneity was obtained from the Cochran’s *Q* statistic (**Supplementary Table S8**).

## 4 Discussion

Contemporary scientific inquiries have illuminated its intricate participation in the onset and advancement of numerous malignancies, such as pancreatic, breast, and hepatocellular carcinomas, where approximately 13% of worldwide cancer cases bear an imprint of microbial influence (14, 50). Of particular interest lies the burgeoning connection between the gut microbiome and lymphoma—a field witnessing considerable exploration. To our knowledge, our investigation stands as one of the pioneering endeavors to methodically appraise the causative link between the gut microbiota and the multifarious forms of lymphoma. Through MR study, harnessing the power of a large-scale GWAS dataset, we strive to bridge a critical knowledge gap within this burgeoning research landscape.

Our MR study identified a total of 35 species of intestinal flora as potentially associated with five subtypes of lymphoma, thus substantiating the critical involvement of specific gut microbiota in the etiology and progression of various lymphoma forms. Unlike traditional observational studies, which often struggle with confounding factors such as diet, age, and gender as well as economic level (51), our MR approach effectively mitigates these influences, thereby enhancing the credibility of the derived conclusions.

Traditional observational research have consistently highlighted correlations between the two—for instance, in a study focusing on adolescent and young adult Hodgkin’s lymphoma (AYAHL) patients, it was evidenced that individuals diagnosed with AYAHL manifested a notably diminished presence of rare gut microbes along with a conspicuously reduced relative abundance of *Actinobacteria* when juxtaposed with their unaffected counterparts (52). Similar findings have been reported in cutaneous T-cell lymphoma (CTCL), where patients typically exhibit a state of gut dysbiosis compared to healthy subjects, a disparity that intensifies as the disease progresses to advanced stages (53). Another study discovered that the intestinal flora of DLBLC patients had a notably increased proportion of *Aspergillus*/*Hypobacterium*, coupled with a decreased representation of butyrate-generating bacterial strains, including *Clostridium*, *Eubacterium*, *Ruminococcus*, and *Roseburia*, compared with that of healthy controls (54). However, it is uncertain whether these, in microbial diversity, act as a risk factor for lymphoma, are caused by the lymphoma itself, or result from the therapeutic interventions that patients receive. Moreover, there is also an association between lymphoma treatment and gut microbes—for instance, in a study of chimeric antigen receptor T cells (CAR-T) and their effectiveness in treating lymphoma found when comparing patient populations experiencing complete *versus* partial remission states, significant temporal disparities were observed in both the biodiversity and relative abundance of key bacterial species, such as *Prevotella*, *Collinsella*, *Bifidobacterium*, and *Sutterella* (55). Cyclophosphamide, a frequently employed chemotherapeutic agent in lymphoma treatment regimens, has been shown to exert a transformative influence on the intestinal microbiome composition of murine models. It notably facilitates the translocation of specific gram-positive bacterial species to secondary lymphoid tissues, concurrently promoting Th17 cells and memory Th1 immune responses and thus contributing to its anti-tumor efficacy (56).

The current research seek to unravel the complex molecular pathways through which gut bacteria influence the progression of lymphoma. Gut microbiota can either activate or detoxify mutagens, which can promote or prevent DNA damage and cancer (57–59)—for example, *H. pylori* increases oxidative stress and can function as an immunogenic stimulus, stimulating persistent immune cell multiplication, which, in turn, leads to lymphoma (60). In addition, gut microbiota have been shown to exert profound influences on the immune response, which can affect lymphocytes. Segmented filamentous bacteria can lead to alterations in T cell activity, often resulting in augmented secretion of cytokines including IFN-γ and IL-10 (61). In murine models, certain bacteria belonging to the *Clostridiales* clusters have been demonstrated to exert a direct influence on T regulatory cell differentiation (62). *Bacterioides fragilis* induces an immune response in Th17 cells, leading to lymphomas (63). In addition, the gut microbiome has systemic effects—for example, Polysaccharide A, a constituent derived from *Bifidobacterium fragilis*, has been shown to stimulate an augmentation in the circulating population of systemic T helper cells (64). Moreover, metabolites produced by the gut microbiome hold a critical position in the lymphoma advancement. The genera *Slackia* and *Lachnospiraceae* abundantly synthesize butyrate *via* several intricate metabolic routes. Empirical evidence suggests that butyrate-producing *Eubacterium* inhibit lymphoma development by attenuating the TNF-activated TLR4/MyD88/NF-κB signaling cascade (65). The above-mentioned genera were also suggested to reduce the risk of lymphoma in our study.

Probiotics constitute a collection of beneficial microorganisms that provide health benefits to the gut through various mechanisms. They achieve this by rectifying intestinal dysbiosis, fostering assimilation, strengthening the mucosal lining, and dampening inflammatory processes (66). Research has shown that *Lactobacillus* is a common probiotic, which inhibits the progression of colorectal cancer by secreting small molecules such as indole-3-lactic acid, downregulating microRNA (miRNA)-155 and upregulating miRNA-26b and miRNA-18a (67, 68). Meanwhile, *Lactobacillus* can produce conjugated linolenic acid in the intestine and secrete extracellular polysaccharides to promote tumor cell apoptosis (69). Some lactobacilli can also improve the content of short-chain fatty acids in intestines, prompting the proliferation of more probiotics and reducing cellular carcinogenesis (70). This finding aligns with our observations suggesting that family *Lactobacillaceae* may reduce the risk of T/NK cell lymphoma. In animal experiments, *Lactobacillus johnsonii*-deficient mice suffering from ataxia–telangiectasia had a higher incidence of lymphoma in a mouse model whose genotoxicity was reduced by a short-term oral administration of *Lactobacillus* (71). Better treatment outcomes and prognosis in DLBCL patients significantly enriched with *Lactobacillus* fermentum have also been observed in clinical trials. Combined with our findings, we can further investigate the protective mechanism of *Lactobacillus* against lymphoma. This may lead to the development of *Lactobacillus*-enriched drugs or genetically engineered supplements to prevent lymphoma.

Family *Bifidobacteriaceae* and order Bifidobacteriaceae belong to the phylum Actinobacteria, which constitute a substantial component of the human gastrointestinal flora (72). Some studies have found health-promoting and anti-tumor effects (73). A study discovered that certain strains of *Bifidobacterium bifidum* in mice reduced tumor load by triggering an anti-tumor host immune response in conjunction with PD-1 blockade or oxaliplatin treatment (74). Bifidobacteria limit the formation of free radicals by binding iron within the colon, which can consequently diminish the risk of colorectal carcinogenesis (75). A study in adolescents found that *Bifidobacterium bifidum* coordinated fibroblasts to inhibit colorectal tumorigenesis through the Wnt signaling pathway (76). However, no studies have investigated whether they may have an impact on lymphoma. Our study revealed that family *Bifidobacteriaceae* and order Bifidobacteriaceae are causally associated with HL disease and may increase the risk of HL. This finding is the first report, which also provides new ideas to further explore the immune mechanism between *Bifidobacteria* and lymphoma in the future.

Genus *Alistipes* represents a relatively novel group within the bacterial domain, and our study found it to be a protective factor in DLBCL and FL. In previous studies, it has been conceptualized as a “double-edged sword”. Research have claimed that A*listipes* has a pathogenic effect on colorectal cancer through the IL-6/STAT3 pathway and has been linked to depression (77). Although a pathogenic role for *Alistipes* has been observed in colorectal cancer, recent studies suggest that it may also have a positive impact on cancer immunotherapy by altering the tumor microenvironment—for example, in patients responding well to nabulizumab for non-small cell lung cancer, there was an increased abundance of *Alistipes* (78). Nevertheless, there is currently no explicit evidence in the existing literature that directly implicates a relationship between *Alistipes* and the development of lymphoma. The immune mechanism between *Alistipes* and lymphoma can be further explored in the future, providing ideas for microbial-assisted therapy.

Genus *Coprobacter* is an important member of the phylum Trichoderma and the principal sources of butyric acid production. Butyrate acts as an energy-producing substance in the colon, stabilizes hypoxia-inducible factor to sustain the characteristic anaerobic milieu within the gut environment, and regulates Claudin-1 and synaptopodin expression to maintain gut barrier integrity. Additionally, it limits the production of inflammation-associated cytokines as well as inhibits oncogenic signaling cascades, including TGF-β and Akt/ERK signaling (79). Genus *Coprobacter* may help to suppress the immune response and alleviate the intensity of allergy, in addition to being associated with depression and language development in young children (80, 81). In our study, genus *Coprobacter* can potentially augment the susceptibility to DLBCL while demonstrating a protective effect against HL; this conclusion this still needs further studies. Butyrate has also been found to enhance cancer treatment efficacy by modulating intracellular calcium, and the role of these butyrate-producing intestinal flora in the development and treatment of lymphomas remains to be further investigated (82).

This MR study benefits from utilizing a vast amount of data from multiple published GWAS summaries and controlling for partial confounding and reverse causality. In addition, the study focuses on GWAS data for gut microbiota and lymphoma confined in European populations, reducing potential biases arising from genetic and environmental heterogeneity across different ethnic backgrounds.

Nevertheless, our study had several limitations. Firstly, it is important to take care when generalizing the study results to other ethnic populations, as the bulk of patients in the combined GWAS dataset comes from European populations. This may introduce biases into the estimations and compromise the universal applicability of the conclusions. Secondly, the impact of gut microbiota appears to exhibit heterogeneity across different pathological subtypes of lymphoma, requiring further investigation into the precise biological mechanisms that underlie the connection between the gut microbiome and the diverse pathological manifestations of lymphoma. Thirdly, gut microbiome GWAS is still in its infancy and the count of relevant loci is comparatively modest relative to lymphoma, and some bacteria may not be adequately characterized at the genus or species level. As the GWAS continues to expand and incorporate larger sample sizes, along with the use of advanced shotgun metagenomic sequencing techniques, there is a promising prospect that more definitive and nuanced features will emerge with greater clarity (83). Fourthly, after implementing the stringent Bonferroni correction to the MR analysis results, the associations in the current study were not statistically significant. Therefore, the findings should only be regarded as suggestive evidence of a potential association. In addition, there are fewer *in vivo* or *in vitro* studies of specific flora associated with lymphoma, which cannot permit adequate comparison and discussion. Subsequent research endeavors can capitalize on these preliminary insights to determine a more definitive link between gut microbiota composition and the etiology of lymphoma. Consequently, conclusions drawn from our current work should be considered provisional rather than conclusive, highlighting the need for further corroboration.

## 5 Conclusion

We assessed the causal link between gut microbiota and lymphoma through MR analysis of public GWAS data, identifying specific bacteria that might contribute to lymphoma risk and potential protective taxa. Our study provides new ideas for possible therapeutic approaches to lymphoma and clues to the pathogenesis of lymphoma.

## Data availability statement

The datasets presented in this study can be found in online repositories. The original contributions presented in the study are included in the article/**Supplementary Material**. Further inquiries can be directed to the corresponding author.

## Ethics statement

Ethical approval was not required for the study involving humans in accordance with the local legislation and institutional requirements. Written informed consent to participate in this study was not required from the participants or the participants’ legal guardians/next of kin in accordance with the national legislation and the institutional requirements.

## Author contributions

BL: Writing – original draft, Writing – review & editing, Data curation, Software. YH: Data curation, Software, Writing – original draft, Writing – review & editing. ZF: Data curation, Writing – original draft. YC: Data curation, Writing – original draft. XG: Data curation, Writing – original draft. SD: Data curation, Writing – original draft. CL: Data curation, Writing – original draft. DW: Writing – review & editing.

## Supplementary material

Supplementary Table S1 SNPs with confounding factors.

Supplementary Table S2 Calculated F-statistics for all IVs.

Supplementary Table S3 MR-Steiger analysis results for all SNPs.

Supplementary Table S4 The IVs after harmonization.

Supplementary Table S5 The results of MR analysis.

Supplementary Table S6 The results of MR-Egger.

Supplementary Table S7 The results of MR-PRESSO text.

Supplementary Table S8 The results of the Cochran’s Q statistic.

## References

[1] Armitage JO Gascoyne RD Lunning MA Cavalli F. Non-hodgkin lymphoma. Lancet. (2017) 390:298–310. doi: 10.1016/S0140-6736(16)32407-2 28153383

[2] Elenitoba-Johnson KSJ Lim MS. New insights into lymphoma pathogenesis. Annu Rev Pathol. (2018) 13:193–217. doi: 10.1146/annurev-pathol-020117-043803 29140757

[3] Sabattini E Bacci F Sagramoso C Pileri SA. WHO classification of tumours of haematopoietic and lymphoid tissues in 2008: an overview. Pathologica. (2010) 102:83–7. 21171509

[4] Bray F Ferlay J Soerjomataram I Siegel RL Torre LA Jemal A. Global cancer statistics 2018: GLOBOCAN estimates of incidence and mortality worldwide for 36 cancers in 185 countries. CA Cancer J Clin. (2018) 68:394–424. doi: 10.3322/caac.21492 30207593

[5] Sung H Ferlay J Siegel RL Laversanne M Soerjomataram I Jemal A. Global cancer statistics 2020: GLOBOCAN estimates of incidence and mortality worldwide for 36 cancers in 185 countries. CA Cancer J Clin. (2021) 71:209–49. doi: 10.3322/caac.21660 33538338

[6] Sergentanis TN Kanavidis P Michelakos T Petridou ET. Cigarette smoking and risk of lymphoma in adults: a comprehensive meta-analysis on Hodgkin and non-Hodgkin disease. Eur J Cancer Prev. (2013) 22:131–50. doi: 10.1097/CEJ.0b013e328355ed08 22759975

[7] Shankland KR Armitage JO Hancock BW. Non-hodgkin lymphoma. Lancet. (2012) 380:848–57. doi: 10.1016/S0140-6736(12)60605-9 22835603

[8] Ingham RR Reagan JL Dalia S Furman M Merhi B Nemr S. The relationship between obesity and lymphoma: A meta-analysis of prospective cohort studies. Blood. (2011) 118:5198. doi: 10.1182/blood.V118.21.5198.5198

[9] Murray PG Young LS. An etiological role for the Epstein-Barr virus in the pathogenesis of classical Hodgkin lymphoma. Blood. (2019) 134:591–6. doi: 10.1182/blood.2019000568 31186275

[10] Yang J Wang P Lv ZB Wei LG Xu YL Zhou A. AIDS-related non-Hodgkin lymphoma: imaging feature analysis of 27 cases and correlation with pathologic findings. Asian Pac J Cancer Prev. (2014) 15:7769–73. doi: 10.7314/apjcp.2014.15.18.7769 25292061

[11] Leuraud K Richardson DB Cardis E Daniels RD Gillies M O’Hagan JA. Ionising radiation and risk of death from leukaemia and lymphoma in radiation-monitored workers (INWORKS): an international cohort study. Lancet Haematol. (2015) 2:e276–81. doi: 10.1016/S2352-3026(15)00094-0 PMC4587986 26436129

[12] Rana I Dahlberg S Steinmaus C Zhang L. Benzene exposure and non-Hodgkin lymphoma: a systematic review and meta-analysis of human studies. Lancet Planet Health. (2021) 5:e633–43. doi: 10.1016/S2542-5196(21)00149-2 PMC9109598 34450064

[13] Zintzaras E Voulgarelis M Moutsopoulos HM. The risk of lymphoma development in autoimmune diseases: a meta-analysis. Arch Intern Med. (2005) 165:2337–44. doi: 10.1001/archinte.165.20.2337 16287762

[14] Uribe-Herranz M Klein-González N Rodríguez-Lobato LG Juan M de Larrea CF. Gut microbiota influence in hematological Malignancies: from genesis to cure. Int J Mol Sci. (2021) 22:1026. doi: 10.3390/ijms22031026 33498529 PMC7864170

[15] Lynch SV Pedersen O. The human intestinal microbiome in health and disease. N Engl J Med. (2016) 375:2369–79. doi: 10.1056/NEJMra1600266 27974040

[16] Scott AJ Alexander JL Merrifield CA Cunningham D Jobin C Brown R. International Cancer Microbiome Consortium consensus statement on the role of the human microbiome in carcinogenesis. Gut. (2019) 68:1624–32. doi: 10.1136/gutjnl-2019-318556 PMC6709773 31092590

[17] Shi Z Zhang M. Emerging roles for the gut microbiome in lymphoid neoplasms. Clin Med Insights Oncol. (2021) 15:11795549211024197. doi: 10.1177/11795549211024197 34211309 PMC8216388

[18] Cullin N Azevedo Antunes C Straussman R Stein-Thoeringer CK Elinav E. Microbiome and cancer. Cancer Cell. (2021) 39:1317–41. doi: 10.1016/j.ccell.2021.08.006 34506740

[19] Upadhyay Banskota S Skupa SA El-Gamal D D’Angelo CR. Defining the role of the gut microbiome in the pathogenesis and treatment of lymphoid Malignancies. Int J Mol Sci. (2023) 24:2309. doi: 10.3390/ijms24032309 36768631 PMC9916782

[20] Kuo SH Wu MS Yeh KH Lin CW Hsu PN Chen LT. Novel insights of lymphomagenesis of helicobacter pylori-dependent gastric mucosa-associated lymphoid tissue lymphoma. Cancers (Basel). (2019) 11:547. doi: 10.3390/cancers11040547 30999581 PMC6520890

[21] Yoon SE Kang W Choi S Park Y Chalita M Kim H. The influence of microbial dysbiosis on immunochemotherapy-related efficacy and safety in diffuse large B-cell lymphoma. Blood. (2023) 141:2224–38. doi: 10.1182/blood.2022018831 36724450

[22] Shi Z Hu G Li MW Zhang L Li X Li L. Gut microbiota as non-invasive diagnostic and prognostic biomarkers for natural killer/T-cell lymphoma. Gut. (2023) 72:1999–2002. doi: 10.1136/gutjnl-2022-328256 36347595 PMC10511952

[23] Stein-Thoeringer CK Saini NY Zamir E Blumenberg V Schubert ML Mor U. A non-antibiotic-disrupted gut microbiome is associated with clinical responses to CD19-CAR-T cell cancer immunotherapy. Nat Med. (2023) 29:906–16. doi: 10.1038/s41591-023-02234-6 PMC10121864 36914893

[24] Jenq RR Ubeda C Taur Y Menezes CC Khanin R Dudakov JA. Regulation of intestinal inflammation by microbiota following allogeneic bone marrow transplantation. J Exp Med. (2012) 209:903–11. doi: 10.1084/jem.20112408 PMC3348096 22547653

[25] Shi Z Li H Song W Zhou Z Li Z Zhang M. Emerging roles of the gut microbiota in cancer immunotherapy. Front Immuno. (2023) 14:1139821. doi: 10.3389/fimmu.2023.1139821 PMC9992551 36911704

[26] Greenland S. An introduction to instrumental variables for epidemiologists. Int J Epidemiol. (2018) 47:358. doi: 10.1093/ije/dyx275 29294084

[27] Emdin CA Khera AV Kathiresan S. Mendelian randomization. JAMA. (2017) 318:1925–6. doi: 10.1001/jama.2017.17219 29164242

[28] Davey Smith G Hemani G. Mendelian randomization: genetic anchors for causal inference in epidemiological studies. Hum Mol Genet. (2014) 23:R89–98. doi: 10.1093/hmg/ddu328 PMC4170722 25064373

[29] Long Y Tang L Zhou Y Zhao S Zhu H. Causal relationship between gut microbiota and cancers: a two-sample Mendelian randomisation study. BMC Med. (2023) 21:66. doi: 10.1186/s12916-023-02761-6 36810112 PMC9945666

[30] Kurilshikov A Medina-Gomez C Bacigalupe R Radjabzadeh D Wang J Demirkan A. Large-scale association analyses identify host factors influencing human gut microbiome composition. Nat Genet. (2021) 53:156–65. doi: 10.1038/s41588-020-00763-1 PMC8515199 33462485

[31] Yuan Z Kang Y Mo C Huang S Qin F Zhang J. Causal relationship between gut microbiota and tuberculosis: a bidirectional two-sample Mendelian randomization analysis. Respir Res. (2024) 25:16. doi: 10.1186/s12931-023-02652-7 38178098 PMC10765819

[32] Chen G Kuang Z Li F Li J. The causal relationship between gut microbiota and leukemia: a two-sample Mendelian randomization study. Front Microbiol. (2023) 14:1293333. doi: 10.3389/fmicb.2023.1293333 38075916 PMC10703164

[33] Codd V Nelson CP Albrecht E Mangino M Deelen J Buxton JL. Identification of seven loci affecting mean telomere length and their association with disease. Nat Genet. (2013) 45:422–427e4272. doi: 10.1038/ng.2528 23535734 PMC4006270

[34] Burgess S Thompson SG CRP CHD Genetics Collaboration. Avoiding bias from weak instruments in Mendelian randomization studies. Int J Epidemiol. (2011) 40:755–64. doi: 10.1093/ije/dyr036 21414999

[35] Verbanck M Chen CY Neale B Do R. Detection of widespread horizontal pleiotropy in causal relationships inferred from Mendelian randomization between complex traits and diseases. Nat Genet. (2018) 50:693–8. doi: 10.1038/s41588-018-0099-7 PMC6083837 29686387

[36] Hemani G Tilling K Davey Smith G. Orienting the causal relationship between imprecisely measured traits using GWAS summary data. PloS Genet. (2017) 13:e1007081. doi: 10.1371/journal.pgen.1007081 29149188 PMC5711033

[37] Burgess S Small DS Thompson SG. A review of instrumental variable estimators for Mendelian randomization. Stat Methods Med Res. (2017) 26:2333–55. doi: 10.1177/0962280215597579 PMC5642006 26282889

[38] Burgess S Butterworth A Thompson SG. Mendelian randomization analysis with multiple genetic variants using summarized data. Genet Epidemiol. (2013) 37:658–65. doi: 10.1002/gepi.21758 PMC4377079 24114802

[39] Bowden J Davey Smith G Burgess S. Mendelian randomization with invalid instruments: effect estimation and bias detection through Egger regression. Int J Epidemiol. (2015) 44:512–25. doi: 10.1093/ije/dyv080 PMC4469799 26050253

[40] Bowden J Davey Smith G Haycock PC Burgess S. Consistent estimation in Mendelian randomization with some invalid instruments using a weighted median estimator. Genet Epidemiol. (2016) 40:304–14. doi: 10.1002/gepi.21965 PMC4849733 27061298

[41] Hartwig FP Davey Smith G Bowden J. Robust inference in summary data Mendelian randomization via the zero modal pleiotropy assumption. Int J Epidemiol. (2017) 46:1985–98. doi: 10.1093/ije/dyx102 PMC5837715 29040600

[42] Lee CH Cook S Lee JS Han B. Comparison of two meta-analysism methods: inverse-variance-weighted average and weighted sum of Z-scores. Genomics Inform. (2016) 14:173–80. doi: 10.5808/gi.2016.14.4.173 PMC5287121 28154508

[43] Ooi BNS Loh H Ho PJ Milne RL Giles G Gao C. The genetic interplay between body mass index, breast size and breast cancer risk: a Mendelian randomization analysis. Int J Epidemiol. (2019) 48:781–94. doi: 10.1093/ije/dyz124 PMC6659372 31243447

[44] Hu J Song J Chen Z Yang J Shi Q Jin F. Reverse causal relationship between periodontitis and shortened telomere length: Bidirectional two-sample Mendelian random analysis. Front Immunol. (2022) 13:1057602. doi: 10.3389/fimmu.2022.1057602 36601105 PMC9806346

[45] Burgess S Thompson SG. Interpreting findings from Mendelian randomization using the MR-Egger method. Eur J Epidemiol. (2017) 32:377–89. doi: 10.1007/s10654-017-0255-x PMC5506233 28527048

[46] Bowden J Del Greco MF Minelli C Davey Smith G Sheehan N Thompson J. A framework for the investigation of pleiotropy in two-sample summary data Mendelian randomization. Stat Med. (2017) 36:1783–802. doi: 10.1002/sim.7221 PMC5434863 28114746

[47] Nazarzadeh M Pinho-Gomes AC Bidel Z Dehghan A Canoy D Hassaine A. Plasma lipids and risk of aortic valve stenosis: a Mendelian randomization study. Eur Heart J. (2020) 41:3913–20. doi: 10.1093/eurheartj/ehaa070 PMC7654932 32076698

[48] Sekula P Del Greco MF Pattaro C Köttgen A. Mendelian randomization as an approach to assess causality using observational data. J Am Soc Nephrol. (2016) 27:3253–65. doi: 10.1681/asn.2016010098 PMC5084898 27486138

[49] Hemani G Zheng J Elsworth B Wade KH Haberland V Baird D. The MR-Base platform supports systematic causal inference across the human phenome. Elife. (2018) 7:e34408. doi: 10.7554/eLife.34408 29846171 PMC5976434

[50] de Martel C Georges D Bray F Ferlay J Clifford GM. Global burden of cancer attributable to infections in 2018: a worldwide incidence analysis. Lancet Glob Health. (2020) 8:e180–90. doi: 10.1016/S2214-109X(19)30488-7 31862245

[51] Davies NM Holmes MV Davey Smith G. Reading Mendelian randomisation studies: a guide, glossary, and checklist for clinicians. BMJ. (2018) 362:k601. doi: 10.1136/bmj.k601 30002074 PMC6041728

[52] Cozen W Yu G Gail MH Ridaura VK Nathwani BN Hwang AE. Fecal microbiota diversity in survivors of adolescent/young adult Hodgkin lymphoma: a study of twins. Br J Cancer. (2013) 108:1163–7. doi: 10.1038/bjc.2013.60 PMC3619077 23443674

[53] Hooper MJ LeWitt TM Pang Y Veon FL Chlipala GE Feferman L. Gut dysbiosis in cutaneous T-cell lymphoma is characterized by shifts in relative abundances of specific bacterial taxa and decreased diversity in more advanced disease. J Eur Acad Dermatol Venereol. (2022) 36:1552–63. doi: 10.1111/jdv.18125 PMC9391260 35366365

[54] Meihong Z. Analysis of intestinal flora and metabolites in patients with primary gastrointestinal diffuse large B-cell lymphoma. Gannan Med Univ. (2023). doi: 10.27959/d.cnki.ggnyx.2023.000326

[55] Hu Y Li J Ni F Yang Z Gui X Bao Z. CAR-T cell therapy-related cytokine release syndrome and therapeutic response is modulated by the gut microbiome in hematologic Malignancies. Nat Commun. (2022) 13:5313. doi: 10.1038/s41467-022-32960-3 36085303 PMC9461447

[56] Viaud S Saccheri F Mignot G Yamazaki T Daillère R Hannani D. The intestinal microbiota modulates the anticancer immune effects of cyclophosphamide. Science. (2013) 342:971–6. doi: 10.1126/science.1240537 PMC4048947 24264990

[57] Reddy BS Mangat S Weisburger JH Wynder EL. Effect of high-risk diets for colon carcinogenesis on intestinal mucosal and bacterial beta-glucuronidase activity in F344 rats. Cancer Res. (1977) 37:3533–6. 908005

[58] Knasmüller S Steinkellner H Hirschl AM Rabot S Nobis EC Kassie F. Impact of bacteria in dairy products and of the intestinal microflora on the genotoxic and carcinogenic effects of heterocyclic aromatic amines. Mutat Res. (2001) 480–481:129–38. doi: 10.1016/S0027-5107(01)00176-2 11506806

[59] Blaser MJ Atherton JC. Helicobacter pylori persistence: biology and disease. J Clin Invest. (2004) 113:321–33. doi: 10.1172/JCI200420925 PMC324548 14755326

[60] O’Rourke JL Dixon MF Jack A Enno A Lee A. Gastric B-cell mucosa-associated lymphoid tissue (MALT) lymphoma in an animal model of ‘Helicobacter heilmannii’ infection. J Pathol. (2004) 203:896–903. doi: 10.1002/path.1593 15258991

[61] Gaboriau-Routhiau V Rakotobe S Lécuyer E Mulder I Lan A Bridonneau C. The key role of segmented filamentous bacteria in the coordinated maturation of gut helper T cell responses. Immunity. (2009) 31:677–89. doi: 10.1016/j.immuni.2009.08.020 19833089

[62] Round JL Mazmanian SK. Inducible Foxp3+ Regulatory T-cell development by a commensal bacterium of the intestinal microbiota. PNAS. (2010) 107:12204–9. doi: 10.1073/pnas.0909122107 PMC2901479 20566854

[63] Wu S Rhee KJ Albesiano E Rabizadeh S Wu X. A human colonic commensal promotes colon tumorigenesis via activation of T helper type 17 T cell responses. Nat Med. (2009) 15:1016–22. doi: 10.1038/nm.2015 PMC3034219 19701202

[64] Mazmanian SK Liu CH Tzianabos AO Kasper DL. An immunomodulatory molecule of symbiotic bacteria directs maturation of the host immune system. Cell. (2005) 122:107–18. doi: 10.1016/j.cell.2005.05.007 16009137

[65] Lu H Xu X Fu D Gu Y Fan R Yi H. Butyrate-producing Eubacterium rectale suppresses lymphomagenesis by alleviating the TNF-induced TLR4/MyD88/NF-κB axis. Cell Host Microbe. (2022) 30:1139–1150.e7. doi: 10.1016/j.chom.2022.07.003 35952646

[66] Fredricks DN. The gut microbiota and graft-versus-host disease. J Clin Invest. (2019) 129:1808–17. doi: 10.1172/JCI125797 PMC6486325 31042160

[67] Gao Y Han T Han C Sun H Yang X Zhang D. Propofol regulates the TLR4/NF-κB pathway through miRNA-155 to protect colorectal cancer intestinal barrier. Inflammation. (2021) 44:2078–90. doi: 10.1007/s10753-021-01485-0 34081253

[68] Heydari Z Rahaie M Alizadeh AM Agah S Khalighfard S Bahmani S. Effects of Lactobacillus acidophilus and Bifidobacterium bifidum Probiotics on the Expression of MicroRNAs 135b, 26b, 18a and 155, and Their Involving Genes in Mice Colon Cancer. Probiotics Antimicrob Proteins. (2019) 11:1155–62. doi: 10.1007/s12602-018-9478-8 30311185

[69] Ren Q Yang B Zhu G Wang S Fu C Zhang H. Antiproliferation Activity and Mechanism of c9, t11, c15-CLNA and t9, t11, c15-CLNA from Lactobacillus plantarum ZS2058 on Colon Cancer Cells. Molecules. (2020) 25:1225. doi: 10.3390/molecules25051225 32182796 PMC7179453

[70] Wang G Yu Y Wang YZ Wang JJ Guan R Sun Y. Role of SCFAs in gut microbiome and glycolysis for colorectal cancer therapy. J Cell Physiol. (2019) 234:17023–49. doi: 10.1002/jcp.28436 30888065

[71] Yamamoto ML Maier I Dang AT Berry D Liu J Ruegger PM. Intestinal bacteria modify lymphoma incidence and latency by affecting systemic inflammatory state, oxidative stress, and leukocyte genotoxicity. Cancer Res. (2013) 73:4222–32. doi: 10.1158/0008-5472.CAN-13-0022 PMC3718495 23860718

[72] Milani C Lugli GA Duranti S Turroni F Bottacini F Mangifesta M. Genomic encyclopedia of type strains of the genus Bifidobacterium. Appl Environ Microbiol. (2014) 80:6290–302. doi: 10.1128/AEM.02308-14 PMC4178644 25085493

[73] Geier MS Butler RN Howarth GS. Probiotics, prebiotics and synbiotics: a role in chemoprevention for colorectal cancer? Cancer Biol Ther. (2006) 5:1265–9. doi: 10.4161/cbt.5.10.3296 16969130

[74] Lee SH Cho SY Yoon Y Park C Sohn J Jeong JJ. Bifidobacterium bifidum strains synergize with immune checkpoint inhibitors to reduce tumour burden in mice. Nat Microbiol. (2021) 6:277–88. doi: 10.1038/s41564-020-00831-6 33432149

[75] Skrypnik K Suliburska J. Association between the gut microbiota and mineral metabolism. J Sci Food Agric. (2018) 98:2449–60. doi: 10.1002/jsfa.8724 28991359

[76] Chen S Fan L Lin Y Qi Y Xu C Ge Q. Bifidobacterium adolescentis orchestrates CD143+ cancer-associated fibroblasts to suppress colorectal tumorigenesis by Wnt signaling-regulated GAS1. Cancer Commun (Lond). (2023) 43:1027–47. doi: 10.1002/cac2.12469 PMC10508156 37533188

[77] Parker BJ Wearsch PA Veloo ACM Rodriguez-Palacios A. The genus alistipes: gut bacteria with emerging implications to inflammation, cancer, and mental health. Front Immunol. (2020) 11:906. doi: 10.3389/fimmu.2020.00906 32582143 PMC7296073

[78] Jin Y Dong H Xia L Yang Y Zhu Y Shen Y. The diversity of gut microbiome is associated with favorable responses to anti-PD-1 immunotherapy in Chinese non-small cell lung cancer patients. J Thorac Oncol. (2019) 14:1378–89. doi: 10.1016/j.jtho.2019.04.007 31026576

[79] Singh V Lee G Son H Koh H Kim ES Unno T. Butyrate producers, “The Sentinel of Gut”: Their intestinal significance with and beyond butyrate, and prospective use as microbial therapeutics. Front Microbiol. (2023) 13:1103836. doi: 10.3389/fmicb.2022.1103836 36713166 PMC9877435

[80] Andreo-Martínez P García-Martínez N Sánchez-Samper EP Martínez-González AE. An approach to gut microbiota profile in children with autism spectrum disorder. Environ Microbiol Rep. (2020) 12:115–35. doi: 10.1111/1758-2229.12810 31713352

[81] Valles-Colomer M Falony G Darzi Y Tigchelaar EF Wang J Tito RY. The neuroactive potential of the human gut microbiota in quality of life and depression. Nat Microbiol. (2019) 4:623–32. doi: 10.1038/s41564-018-0337-x 30718848

[82] Che Y Chen G Guo Q Duan Y Feng H Xia Q. Gut microbial metabolite butyrate improves anticancer therapy by regulating intracellular calcium homeostasis. Hepatology. (2023) 78:88–102. doi: 10.1097/HEP.0000000000000047 36947402

[83] Thomas H. Mendelian randomization reveals causal effects of the gut microbiota. Nat Rev Gastroenterol Hepatol. (2019) 16:198–9. doi: 10.1038/s41575-019-0133-y 30850821

