# Supplementary material for: The causal relationship between gut microbiota and lymphoma: a two-sample Mendelian randomization study
Source: Front Immunol. 2024 May 7;15:1397485. doi: 10.3389/fimmu.2024.1397485 (PMC11106390; doi:10.3389/fimmu.2024.1397485)
Supplement: Supplementary file 2 [file DataSheet_2.docx]

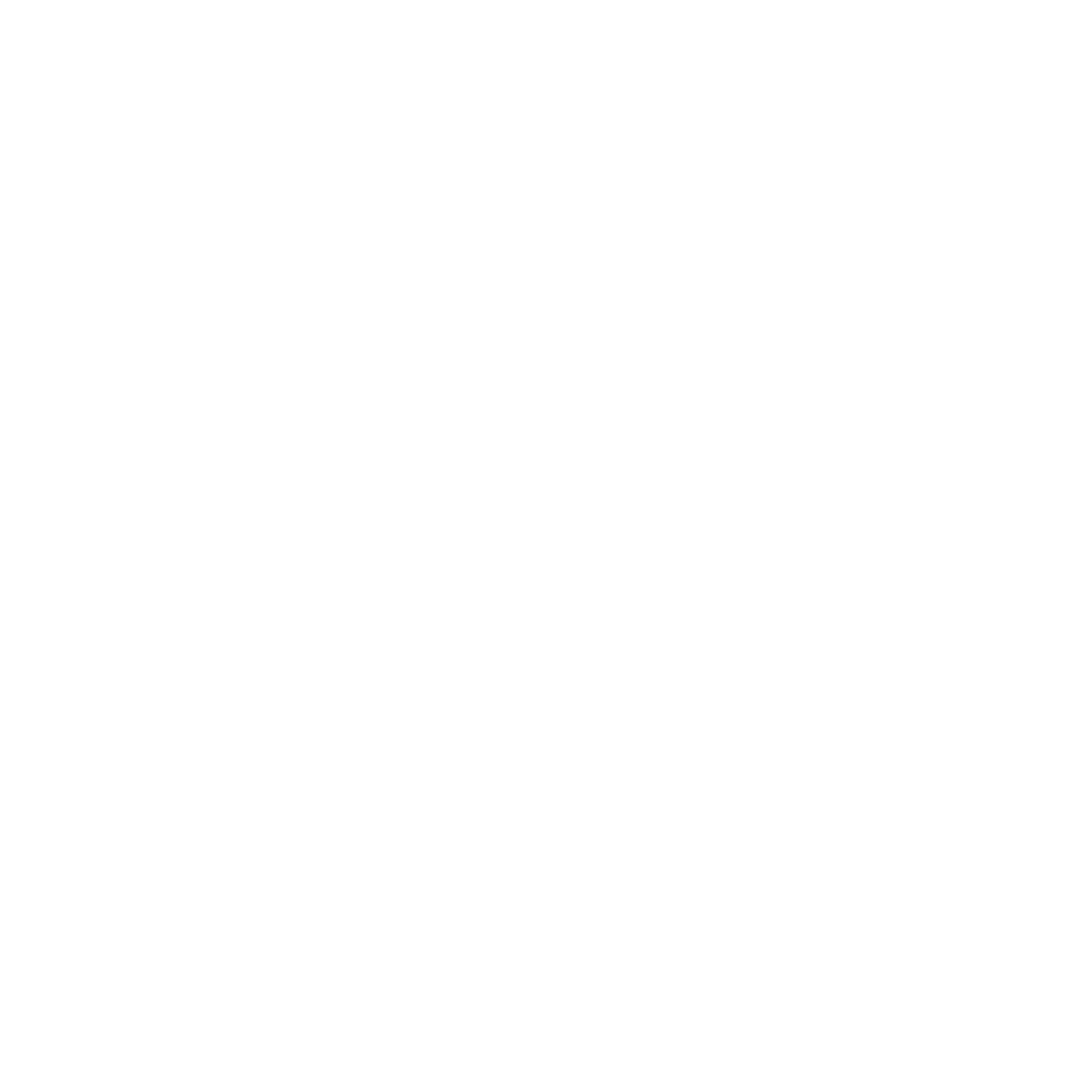


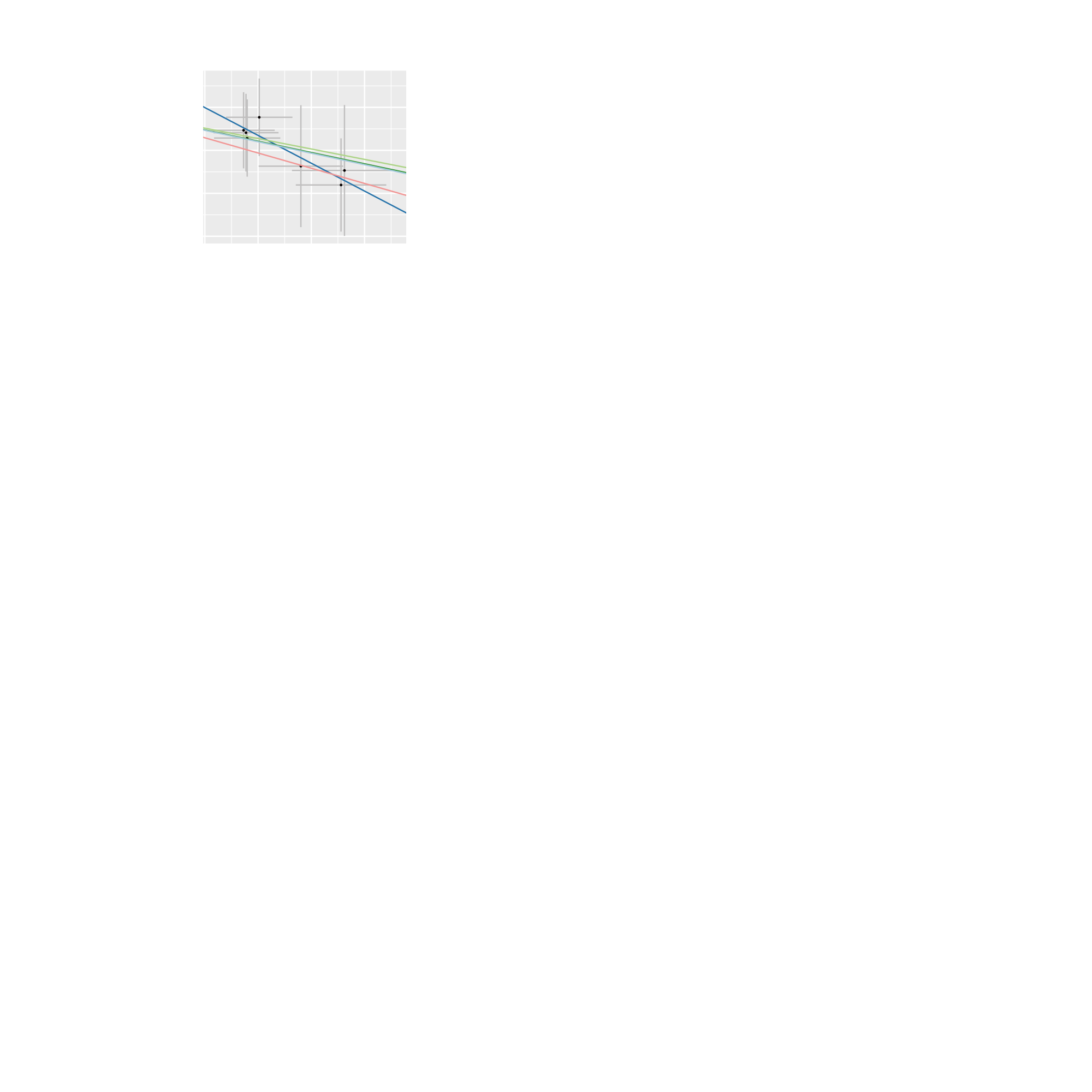


Figure S1 (B) The scatter plots of causal effect for gut microbiota on DLBCL

1. The scatter plots of causal effect for gut microbiota on other and unspecified types of NHL


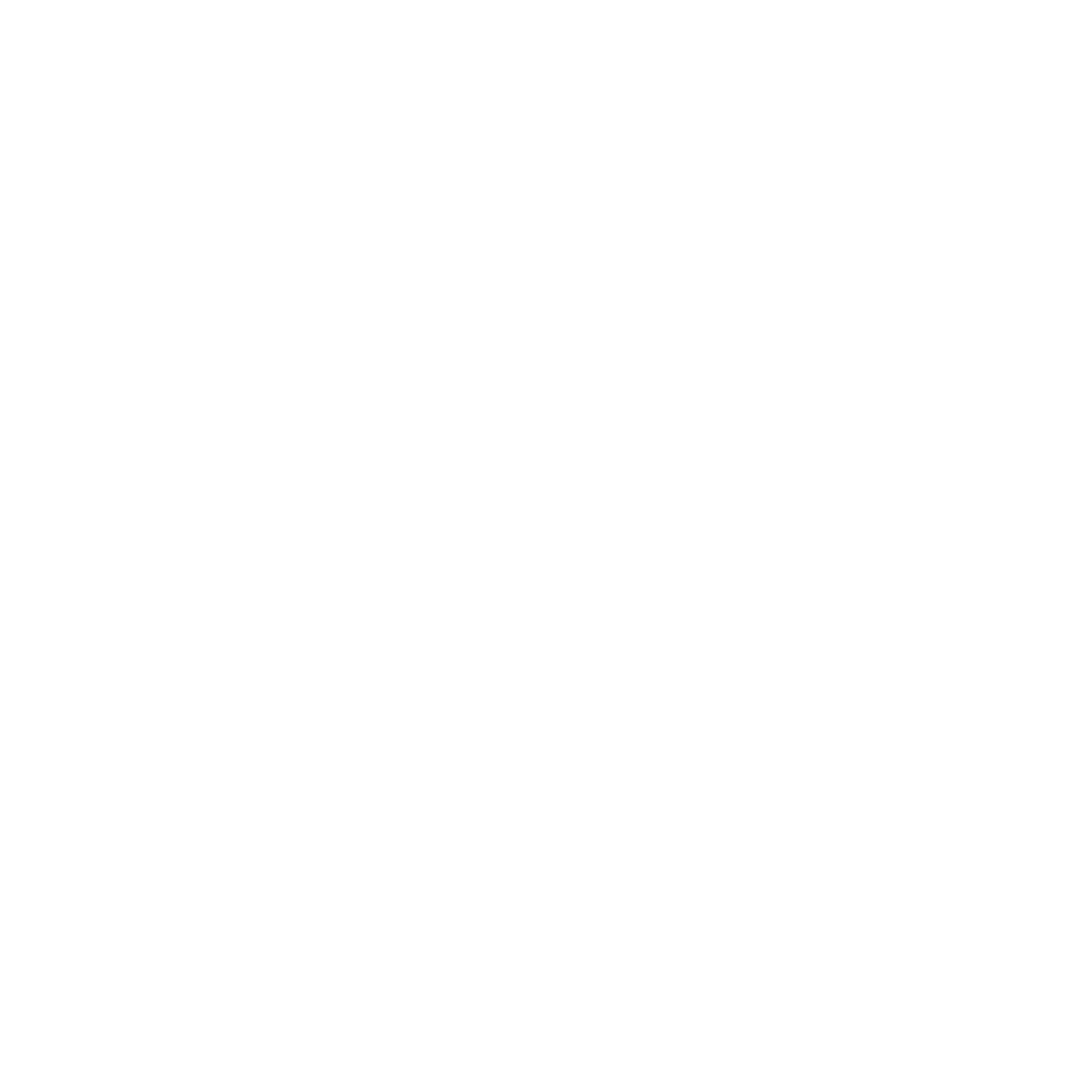
Figure S1 (D) The scatter plots of causal effect for gut microbiota on FL


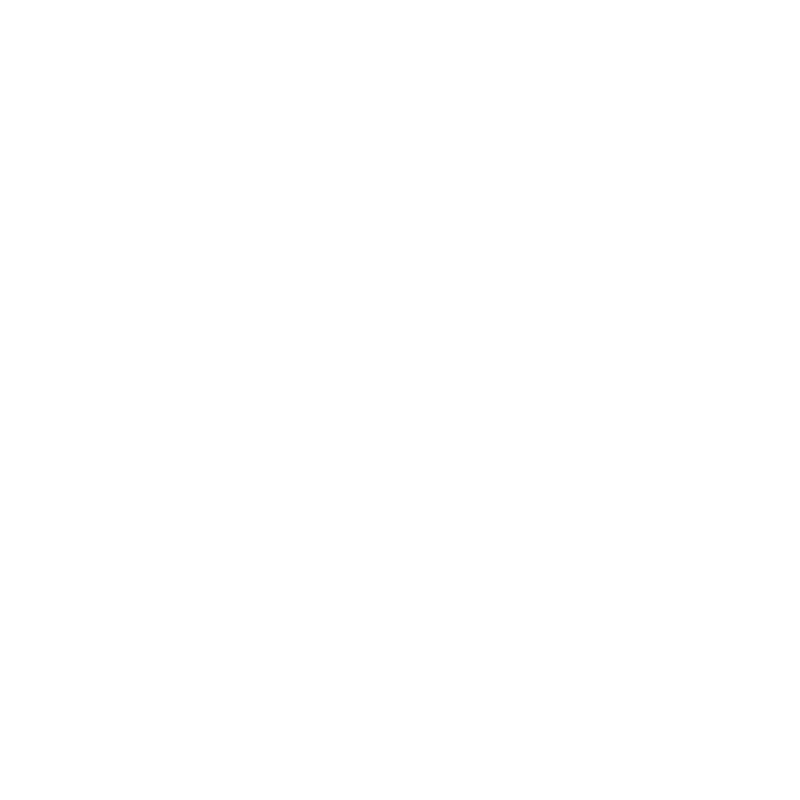
Figure S1 (E) The scatter plots of causal effect for gut microbiota on T/NK cell lymphoma


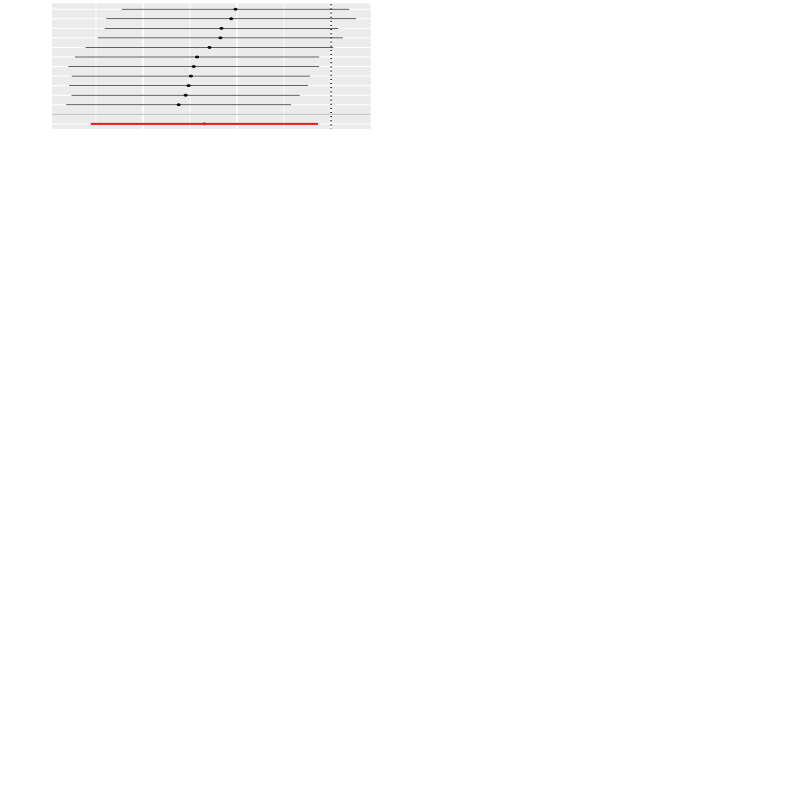
Figure S2 (A) The leave-one-out plots of causal effect for gut microbiota on HL


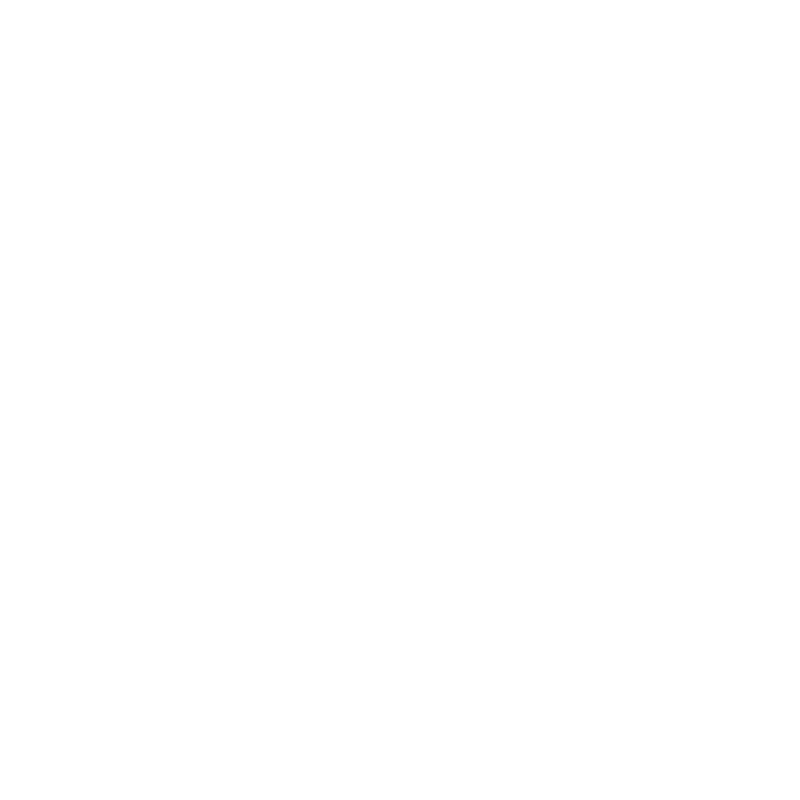
Figure S2 (B) The leave-one-out plots of causal effect for gut microbiota on T/NK cell lymphoma


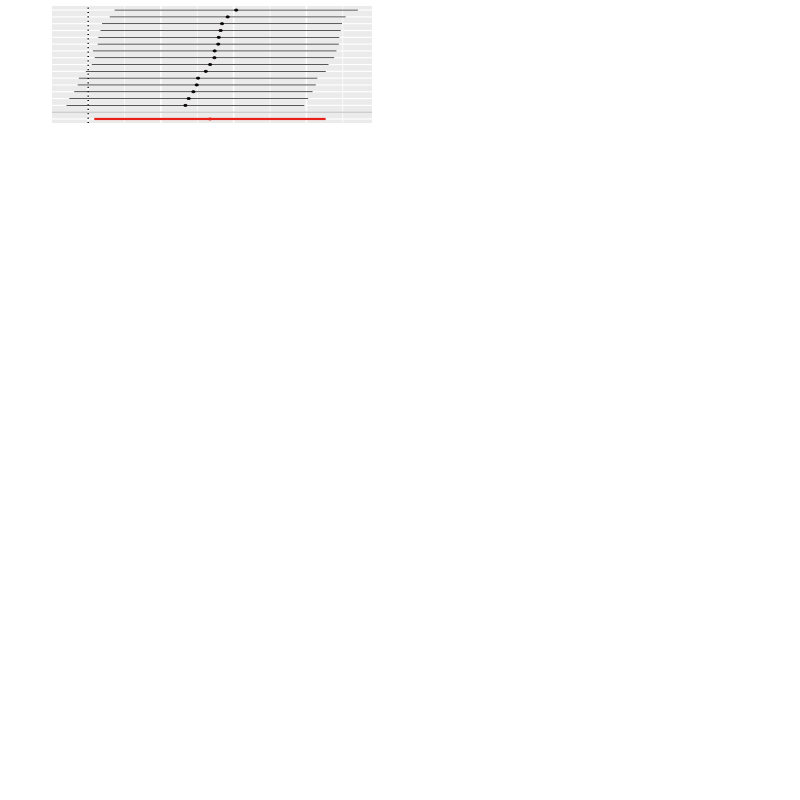
Figure S2 (C) The leave-one-out plots of causal effect for gut microbiota on FL


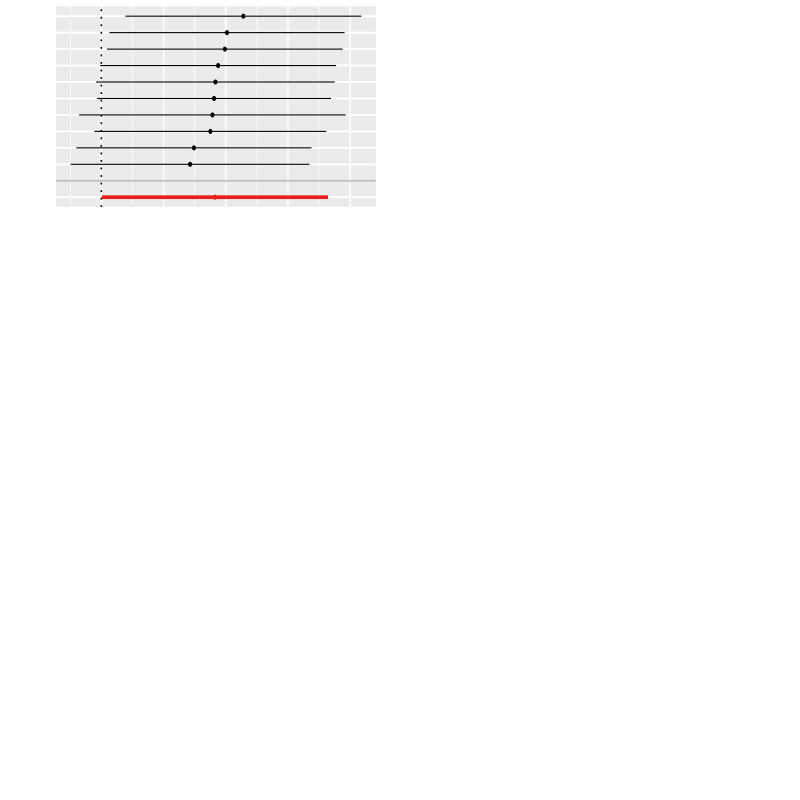
Figure S2 (D) The leave-one-out plots of causal effect for gut microbiota on DLBCL


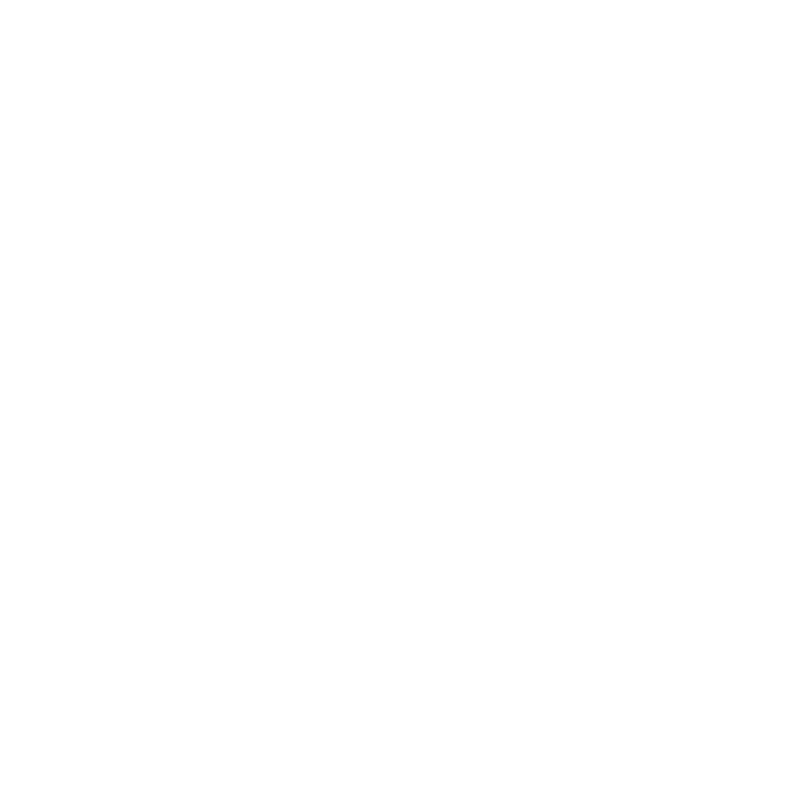
Figure S2 (E) The leave-one-out plots of causal effect for gut microbiota on other and unspecified types of NHL
